# Supplementary material for: Are carbon emissions trading and green financial instruments synergistic? -Comprehensive quantitative research based on content analysis
Source: PLoS One. 2024 Mar 7;19(3):e0298601. doi: 10.1371/journal.pone.0298601 (PMC10919601; doi:10.1371/journal.pone.0298601)
Supplement: S1 Data — (ZIP) [file pone.0298601.s001.zip › Data/地级市Z_F工作报告.zip!/╣π╢1⁄2/╜¡├┼╩╨/└┤╫╘╣1⁄2╓┌║┼í╛┬φ┐╦╩2╛▌═°í┐_2010.docx]

　各位代表：

　　我代表市人民政府向大会报告政府工作，请予审议，并请市政协各位委员和其他列席人员提出意见。

　　2009年工作回顾

　　全年完成生产总值1355.26亿元（初步统计数），增长10.8%，高于全省平均水平；

　　人均生产总值32600元，增长10.4%；

　　地方财政一般预算收入83.63亿元，增长11.98%；

　　外贸进出口总额达110.4亿美元，降幅由年初的28.66%收窄至15.99%；

　　社会消费品零售总额574.34亿元，增长17.4%；

　　实现规模以上工业增加值700.75亿元，增长12.3%；

　　综合交通一体化规划取得初步成果，在推进珠三角一体化中位居前三名；

　　全市各级财政“三农”投入32亿元，增长15.16%；

　　各级财政继续加大民生支出，共计投入45.7亿元，增长22.5%；

　　城镇失业人员实现再就业4.3万人，全市高校毕业生就业率超90%。

　　2009年主要工作回顾

　　刚刚过去的2009年，是我市积极应对国际金融危机，保增长、保民生、保稳定工作取得显著成绩的一年。面对新世纪以来最为困难的经济形势，市政府在省委、省政府和市委的正确领导下，在市人民代表大会及其常委会和市政协的监督支持下，以科学发展观为指导，以实施《珠江三角洲地区改革发展规划纲要》为主轴，扎实推进“三促进一保持”，千方百计保障和改善民生，全市经济运行企稳回升，社会各项事业发展良好，较好地完成了市十三届人大四次会议确定的目标任务。

　　——经济增长好于预期。

　　全年完成生产总值1355.26亿元（初步统计数），增长10.8%，高于全省平均水平，提前一年完成“十一五”经济增长目标。人均生产总值32600元，增长10.4%。地方财政一般预算收入83.63亿元，增长11.98%。

　　——结构调整步伐加快。

　　三次产业结构由2008年的8.4︰58.1︰33.5调整为2009年的7.8︰57.4︰34.8。高新技术产品产值占工业总产值的比重由上年的32%提高到34%；核能、风电、绿色（半导体）光源、医药、船舶等先进制造业快速发展；现代农业建设加快推进，农业增加值增长4%。单位生产总值能耗下降5%。

　　——区域协调发展实现新突破。

　　实施《规划纲要》开局良好，与广佛、珠中及港澳合作全面展开。市区一体化稳步推进。四市发展提速明显，发展差距缩小。

　　——人民生活持续改善。

　　城镇居民人均可支配收入19004元，农村居民人均纯收入7534元，扣除物价因素，分别实际增长12.7%和12.8%。城镇登记失业率控制在2.42%以内。全市参加企业职工基本养老保险缴费人数比上年增加2.23万人。新型农村合作医疗参合率扩大到99.4%，城镇居民基本医疗保险实现全覆盖，开展两险一体化试点，保障水平提高。教育、卫生、文化、体育等社会事业蓬勃发展，基本公共服务均等化积极推进，社会保持和谐稳定。

　　——对口支援四川汶川雁门乡恢复重建工作提前完成。

　　落实对口援建专项资金2.12亿元，建设援建项目51个，以全省第二的速度提前完成省下达我市的援建任务。

　　过去一年，我们主要抓了以下工作：

　　（一）全力保增长。

　　健全重大项目推进机制，抓好减轻企业负担等政策落实，出台重点园区工业投资项目“零收费”、“以商引商”和促进房地产发展等鼓励投资政策。完成固定资产投资492.07亿元，增长25.3%，增幅创近16年新高，全市省重点项目建设进度及电网建设投资额位居全省第一；实际吸收外商直接投资10.37亿美元，增长13.07%，高于全省平均水平；滨江新区建设全面铺开，完成投资7亿多元，道路、水利等13项基础设施工程开工。开展城中村改造试点工作。争取商业银行1540亿元授信支持，落实贷款634亿元。出台扶持外贸出口政策，积极组织企业参加各类促销会展，成功举办摩博会、传统家具精品鉴赏会和首届进出口商品展销会，外贸进出口总额达110.4亿美元，降幅由年初的28.66%收窄至15.99%。积极落实家电、汽车摩托车下乡和家电、汽车以旧换新政策，直接拉动内需3亿元。房地产市场畅旺，全市商品房销售面积349.64万平方米，增长54.6%。社会消费品零售总额574.34亿元，增长17.4%。居民消费价格总水平下降2%。

　　（二）大力推进《规划纲要》实施。

　　加快推进与珠三角核心城市的交通一体化进程，江门市综合交通一体化规划取得初步成果，广珠轻轨江门支线、江肇高速和佛开高速扩建江门段、雁山互通立交、东海路至南环路延伸线完成年度投资计划，广珠铁路江门段、崖门5000吨级航道整治工程建设加快推进，鹤南西江大桥获省立项批复，江门至番禺高速公路及江珠高速北延线获省批准开工建设，江罗高速、江珠高速与港珠澳大桥连接线、中开高速及新会支线等交通项目前期工作进展顺利，广佛江珠轻轨及江恩轻轨、崖门全潮3万吨级航道整治工程开展前期工作，广州南沙至江门铁路进入可研论证阶段。深入推进珠中江区域合作，17个专业领域合作全面开展，珠中江三市跨市公交线路开通，在珠三角率先实现珠中江三市年票互认；实施西江水环境共治，完成市区21家污染企业搬迁。主动融入广佛经济圈，与广州、佛山分别签订了加强重点领域合作和推进一体化合作协议。实现住房公积金珠三角八市互贷。加强与港澳、新加坡合作，与香港四大商会、香港贸发局、香港品质保证局、香港生产力促进局、澳门贸易投资促进局、新加坡中华总商会建立了战略合作关系，开通澳门虚拟机场和江门直达香港、澳门旅游直通车，推进“世界文化遗产两日游”。加大市区一体化推进力度，实现市域年票制、社会保障卡“一卡通”和出租车三区通行，启动失业、工伤、生育保险市级统筹。在《南方日报》公布的《2009年度珠三角竞争力排行榜》中，江门在推进珠三角一体化中位居前三名。

　　（三）提升经济发展质量。

　　扎实推进“四大百亿工程”，实现规模以上工业增加值700.75亿元，增长12.3%。大力提升自主创新能力，新增2个国家级产业基地、1个省级专业镇、10个省市级工程研发中心、4个中国驰名商标、1家出口免验企业、6个省名牌产品、14个省著名商标，获批筹建3个省级检验站。加快传统产业转型升级步伐，技改投资增长35.1%，新认定国家级高新技术企业31家，新增民营科技企业40家，恩平市纺织工业基地和沙湖新型建材工业城列入省十大工业调整和振兴规划。整合财政专项资金支持企业发展，出台绿色（半导体）光源产业政策，大力支持新兴产业发展和重点项目建设，省清洁能源（核电）装备产业园、轨道交通装备制造等大项目招商取得实质性突破，台山核电一期正式动工，上川岛风电、信义特种玻璃、锦兴纺织、富华重工、华泰纸业、亚太纸业等一批投资超10亿元的工业项目建设进展顺利。加大产业园区建设力度，江门产业转移工业园成功竞争成为省示范性产业转移工业园并获得5亿元扶持资金，在建及已建项目24个，总投资17亿元。第三产业对经济增长的贡献率提高14个百分点。金融服务业发展加快，实现增加值39.34亿元，增长26.3%；金融机构贷款余额增长42.9%，增幅创历史新高，存贷比增至44.7%，比上年提高7.6个百分点；金融生态环境明显改善，恩平汇丰村镇银行、民生银行江门支行以及3家小额贷款公司开业运营，新增2家证券公司和1家保险机构进驻我市。企业上市工作步伐加快，1家企业完成上市辅导验收，新增1家企业进入上市辅导期。大力发展现代物流业，实现增加值185亿元，增长15.6%。成功举办2009中国（江门）侨乡旅游节，旅游业保持快速增长，实现总收入104.02亿元，增长14.84%；旅游外汇收入4.1亿美元，增长4.5%。提高节约集约发展水平，关停小火电5万千瓦、小水泥90万吨。大力实施减排工程，完成减排项目76个；新建成10家污水处理厂，全市城镇生活污水处理率由上年的52%提高到60%以上。土地利用管理工作获省综合考评第一，开发利用园地山坡地补充耕地2.6万亩，广海湾处置香港拆建物料新增填海面积1500亩。新会区成为国家可持续发展实验区。

　　（四）扎实推进“三农”工作。

　　全市各级财政“三农”投入32亿元，增长15.16%。农业产业化水平进一步提高，新增9家省市级农业龙头企业、38家农民专业合作经济组织。农业产业化组织带动35万农户，占全市农户数的49.3%，比上年增加3万户，户均增收1960元。加快普及农业科技应用，引进农业新品种70个，推广新技术42项，全市50%以上的行政村和农村经济合作组织建成农村信息直通车服务站。加大农业农村基础设施建设力度，城乡水利防灾减灾工程成效明显，省人大机电排灌议案通过验收，完成农田整治6.9万亩，解决了5.88万农村人口饮水安全问题。深入实施“千企扶千村”工程，免费培训农村劳动力4.5万人，向二、三产业转移农村富余劳动力3.92万人。扎实推进村庄整治。全面铺开集体林权制度改革。海洋与渔业经济实现新发展，总产值突破200亿元，增长32.62%。成功申报“中国锦鲤之乡”。

　　（五）致力改善民生。

　　在财政收入压力较大的情况下，各级财政继续加大民生支出，共计投入45.7亿元，增长22.5%。以增加就业为重点，全市新安排就业5.3万人，城镇失业人员实现再就业4.3万人，全市高校毕业生就业率超90%。实施全民技能提升计划，开展各类职业培训7.1万人。扩大社会保障覆盖面，提高保障水平，增加企业退休人员养老保险金，2.3万被征地农民纳入养老保险试点，市区实行最低生活保障分类施保、家庭病床医疗费纳入基本医疗保险范围、部分大病门诊医疗费用纳入统筹基金按住院标准支付。残疾人工作迈入全省先进行列。加大教育强市建设力度，全市各级财政教育投入21亿元，增长12.43%，完成2个教育强市（县）和21个教育强镇（街）的申报工作；实施义务教育学校绩效工资，落实中小学教师工资福利待遇“两相当”，稳妥解决代课教师问题。加强公共卫生应急能力建设，有效防控甲型H1N1流感等重大疫病。完成省下达的人口计生指标，蓬江区、新会区荣获“全国计划生育优质服务先进区”称号。推进住房保障体系建设，廉租住房和经济适用住房保障家庭分别新增554户和189户。做好住房公积金缴存扩面工作，提高住房公积金最高贷款限额，实现跨行按月自动提取还贷。完成农村800户泥砖房和华侨农场1730户危房改造任务。扎实推进创建全国文明城市工作。顺利通过国家园林城市复检。成功举办广东省第二届体育大会、庆祝新中国成立60周年系列活动、第六届侨乡书香节和市树评选活动。“义工”活动、“一元爱心日”、扶助贫困家庭等慈善活动蓬勃开展。加强基层综治信访维稳中心建设，信访工作保持总体平稳向好态势。大力推进平安和谐社区建设，“六好”平安和谐社区由上年的106个增至155个。开展城市综合管理规范化试点工作，在解决占道经营方面取得明显成效。侨务、外事、港澳、对台、民族、宗教、老龄、统计、审计、新闻、出版、档案、方志、气象等工作进一步加强。

　　（六）强化服务型政府建设。

　　开展深入学习实践科学发展观活动，形成应对国际金融危机、推动科学发展的强大动力。按照省的统一部署，完成市、县两级政府机构改革，市政府工作部门由36个减到32个。进一步扩大县级管理权限，减少行政审批事项，把85%的审批权下放各市、区。新行政服务中心投入使用，涉企审批全部进入中心办理，行政审批进一步提速；实行网上审批服务的政府部门扩大到80个，审批事项和网上服务流程分别增至330个和758个。自觉接受市人大及其常委会的监督和政协的民主监督，主动配合做好“人大代表约见市长”工作，共办理人大代表建议138件、政协提案141件，满意率分别达97.06%和99.29%。坚持市政府常务会议学法制度。密切与各民主党派、工会、共青团、妇联等的联系，广泛听取社会各界意见。持续推进ISO质量管理工作。机关作风建设年活动取得成效，开展明察暗访，被暗访到存在问题的7个部门一把手向社会进行公开道歉。实现行政办公经费五个“零增长”。推进廉政建设，惩防腐败体系进一步完善。加大产品质量和食品安全监管整治以及打传、打假、打私力度，市场秩序进一步规范。全市安全生产总体状况良好，各类事故稳中有降。国防建设和民兵预备役工作得到加强，人防“101”工程建设全面完成。

　　各位代表!过去一年，我们经受了国际金融危机的重大挑战，全市经济社会发展取得这样的成绩，确实来之不易。我们深深地体会到，全面落实中央和省的决策部署、结合江门实际创造性地开展工作，是我们应对危机、促进发展的致胜法宝；全市上下众志成城、群策群力、团结奋斗，是我们战胜困难的强大动力。在此，我代表市人民政府，向全市广大工人、农民、知识分子、干部职工和外来建设者，向驻我市人民解放军、武警官兵、人民警察和各民主党派、人民团体、社会各界人士，致以崇高的敬意！向长期关心支持我市发展的港澳台同胞、海外侨胞以及国际友人表示衷心的感谢！

　　去年底开展的“政府工作大家评”问卷调查活动，反映出政府工作与市民群众的期望还有一定的距离，政府工作还有很多需要改进的地方。我们必须清醒地认识到，在国际金融危机的冲击下，长期制约我市经济社会发展的产业集聚水平不高、自主创新能力和产业竞争力不强、城乡区域发展差距较大等深层次矛盾和问题更加突出，外贸进出口降幅大于全省平均水平，财政收支平衡压力较大，保障民生任务艰巨，营商环境有待进一步优化，市民群众对社会治安、城市管理、环保设施建设、机关作风建设等方面的工作满意度不高。对政府工作存在的问题，我们要以对人民高度负责的态度，切实加以解决。

　　2010年工作安排

　　今年经济社会发展预期目标

　　生产总值增长11%；

　　人均生产总值增长10%；

　　全社会固定资产投资增长20%；

　　规模以上工业增加值增长12%；

　　进出口总额增长6%；

　　地方财政一般预算收入增长10.5%；

　　社会消费品零售总额增长16%，居民消费价格涨幅控制在3%左右；

　　服务业增加值占生产总值比重达36.5%；

　　全社会R＆D投入占生产总值比重达1.3%；

　　城镇登记失业率控制在3.3%以内；

　　人口自然增长率控制在6.8％。以内；

　　城镇居民人均可支配收入增长9%，农村居民人均纯收入增长8%。

　　2010年是全面实现“十一五”规划目标、规划“十二五”时期经济社会发展的关键一年。当前全球经济进入复苏期，我国经济企稳向好的势头持续，但同时也面临着发达国家市场需求恢复缓慢、我国经济发展内生动力不足等困难。我们既要保持经济增长的连续性，又要抓住机遇，为今后长远发展优结构、上水平打下坚实基础。

　　今年市政府工作的总体要求是：以邓小平理论和“三个代表”重要思想为指导，深入贯彻落实科学发展观，认真贯彻落实党的十七大及历次全会、省委十届六次全会和市委十一届七次全会精神，全面实施《珠江三角洲地区改革发展规划纲要》，大力推进“三促进一保持”，加快转变发展方式，着力优化经济结构，加强以保障和改善民生为重点的社会建设，全面完成“十一五”规划各项目标任务，推动全市经济社会持续健康较快发展，为实施“十二五”规划奠定良好基础。

　　今年全市经济社会发展的主要预期目标是：生产总值增长11%；人均生产总值增长10%；单位生产总值能耗下降、二氧化硫和化学需氧量排放总量减排完成省下达的“十一五”目标；全社会固定资产投资增长20%；规模以上工业增加值增长12%；进出口总额增长6%；地方财政一般预算收入增长10.5%；社会消费品零售总额增长16%；居民消费价格涨幅控制在3%左右；服务业增加值占生产总值比重达36.5%；全社会R＆D投入占生产总值比重达1.3%；城镇登记失业率控制在3.3%以内；人口自然增长率控制在6.8％。以内；城镇居民人均可支配收入增长9%，农村居民人均纯收入增长8%。

　　为实现上述目标，必须做好以下六个方面工作：

　　一、着力扩内需、增出口，增强经济持续较快发展动力

　　加快基础设施建设。今年安排市重点项目152项，年度投资405.14亿元，比上年增长43.14%。全力配合广珠轻轨江门支线、广珠铁路江门段、广州南沙至江门铁路、江肇高速、佛开高速扩建、江罗高速、江珠高速与港珠澳大桥连接线建设。加快推进广佛江珠轻轨及江恩轻轨、江门至番禺高速公路及江珠高速北延线、中开高速及新会支线、鹤南西江大桥、西环路隧道、新台高速南延线的前期工作，争取提前动工。加快崖门5000吨级航道整治工程建设。大力推进台山核电、台山电厂二期、新会双水发电厂热电联供机组、新会LNG发电厂首期、川岛风电等能源项目建设。加快城乡水利防灾减灾、病险水库除险加固、新增中央投资水利工程等项目建设。抓好小型水库、小堤围、机电排灌、灌渠等小型农田水利基本建设。建设新会崖门国家一级渔港。

　　大力推进城镇化。发挥规划龙头作用，大幅提高控制性详规覆盖率。以旧城镇、旧厂房、旧村庄改造为重点，制定“三旧”改造规划及年度实施计划，大力推进“三旧”改造工作。规划建设市域绿道网，优化人居环境。基本解决城市规划区范围内问题楼盘、问题地块等历史遗留问题。落实农村居民转移落户城镇的政策，进一步提高城镇化水平。扩大廉租住房和经济适用住房保障范围，将市区保障标准由人均住房建筑面积10平方米提高到12平方米。加大廉租住房和经济适用住房建设力度，市级财政安排廉租住房建设资金3035万元，比上年增长95.05%。完善住房公积金制度，提高缴存标准和覆盖面。扶持发展建筑业，力争用2—3年时间振兴江门建筑业。加快无线城市建设，实现无线宽带网络覆盖市区政务、商务重点区域以及核心园区。

　　积极开拓国内外市场。加大开拓国际市场的力度，继续抢抓出口订单。积极与港澳贸发机构、境外商贸协会等合作开展商务交流活动，鼓励企业积极参加国际专业会展，办好珠中江进出口商品展销会，完善投资贸易推介服务平台。积极实施出口市场多元化战略，深度开发欧美及香港等传统市场，大力开拓东盟、俄罗斯、南美、非洲等新兴市场，支持企业走出去设立营销中心。推进加工贸易转型升级，鼓励企业开展自主出口品牌建设，创出口名牌。推动江门名优产品开拓国内市场，抓好以“万村千乡”市场工程为主的农村商业网点建设，落实家电、汽车摩托车下乡和家电、汽车以旧换新工作，促进住房、机动车、旅游和教育文化消费。加强食品药品安全监管。

　　二、着力提升自主创新能力和产业竞争力，加快构建现代产业体系

　　狠抓重点产业和重大项目招商。着力发展装备制造业，改造提升传统产业，培育战略性新兴产业，突出抓好核电装备制造、轨道交通装备制造、绿色（半导体）光源、汽车零部件、石化、造纸、新能源、新材料等重点产业和重大项目的招商。加强对重点地区招商，深化与港澳有关商会和机构的合作，办好粤台经贸交流会、摩博会等重大招商活动。进一步优化营商环境，强化招商引资“洼地”效应。加大以商引商、产业招商和专业招商力度，完善重大项目招商数据库，在引进大型国企、跨国公司上实现新突破。鼓励企业增资扩产；对引进项目全程跟踪服务，提高合同资金到位率，力争实际利用外资增幅高于全省平均水平。

　　大力推进产业集聚发展。落实工业产业布局与发展规划，整合优化全市现有各类工业园区，形成同类或关联企业集群共生、联动发展的产业格局。重点抓好市高新区、江沙工业走廊、银洲湖园区、广海湾园区、江门产业转移工业园，以及江门市先进制造业江沙示范园、鹤山工业城等核心工业园区的建设。抓好15个国家级产业基地、6个省级产业基地、4个省产业集群升级示范区建设，促进现有优势产业集群进一步壮大。完善促进民营经济发展的环境，大力扶持中小企业发展，加快推进中小企业上市，向非公有制经济开放法律没有禁止的各个领域，激活民间投资。

　　切实提高自主创新能力。落实自主创新总体规划，加大各级财政投入，建立鼓励企业加大研发投入的机制。实施创新和科研团队引进计划，完善引进高层次人才政策，设立“江门侨乡杰出专家奖”，重奖作出突出贡献的优秀人才。把加快传统产业转型升级、扶强做大行业内领先的优势产业、提高高新技术产品产值占工业总产值的比重作为重点，明确任务，

　　□ 江门市市长 王南健

　　紧接A02版

　　强化措施，落实责任。实施传统产业科技计划项目78项，实施技术改造项目150项，新培育25家以上高新技术企业，推动35家以上传统企业向科技型企业转型，计算机辅助设计和辅助制造技术应用率达到80%以上。加强科技创新服务平台建设，争取国家级摩托车检验检测中心通过验收，筹建国家机械装备检测重点实验室，加快绿色（半导体）光源、制浆造纸、电声产品等省级检测站建设并争取成为国家级检测中心。鼓励企业和高校建立工程技术研发中心和申报组建省和国家重点实验室，培育创建企业工程研发中心40家以上。积极组织企业参与省部产学研战略联盟。办好科技企业孵化器，建立各类科技孵化器6家以上，并全面运营招商。大力实施专业镇技术创新试点工程。加快市高新区“二次创业”步伐，争取成为国家级高新技术开发区。加强知识产权保护和科技创新环境建设，支持企业实施品牌战略和技术标准战略。

　　大力发展现代服务业。提高现代服务业在国民经济中的比重。重点抓好金融服务业、物流业和旅游业发展。加快社会征信体系建设，继续鼓励在我市新设金融机构，发展信用担保机构，着力打造一流的金融生态环境。制定现代物流业发展规划（2010-2020年），推进南新区、新会银洲湖和鹤山（沙坪-桃源）等现代物流基地建设，抓好保税物流中心、保税仓、出口监管仓等各类保税区域、场所的配套建设，推动传统交通运输仓储企业向专业物流企业转变，力争物流增加值占全市GDP的比重达到14%。打造国内一流旅游胜地，创新旅游主题和线路，不断开拓旅游市场，提升与港澳台、外省及国际旅游合作水平，打响碉楼、温泉、海滨“三张牌”，保持旅游业强势增长。建设市区新的会展中心，抓好绿色光源、麦克风、水暖卫浴等专业市场建设，扩大江门传统家具精品鉴赏会、开平牛仔服装节、水口水暖卫浴展销洽谈会、恩平麦克风及电声视频产品国际展销会等会展品牌影响力。实施核心城区“退二进三”战略，加快推进蓬江区中央商务区建设。

　　三、着力落实《规划纲要》，打造区域发展新优势

　　深化重点领域改革。推进富县强镇事权改革，扩大县级及具备一定人口规模和经济实力的中心镇的管理权限，强化县、镇的社会管理、公共服务和市场监管职责。完善大部门体制改革，完成好省赋予我市的创新审批方式改革试点任务。行政许可部门原则上都要进入行政服务中心，实行“一站式”服务；全面推行网上审批、并联审批。推进事业单位人事制度改革。深化公共财政体制改革，加快构建以基本公共服务均等化为导向的财政投入及保障机制。加快文化体制改革，重点抓好广电网络和新华书店的重组。改革户籍制度，逐步使农民工在住房租赁、医疗卫生、子女教育、社会保障和基本公共服务等方面与城镇居民享有同等待遇。

　　积极融入珠三角一体化。坚持右联珠中、左接广佛，落实好签订的各项合作框架协议，加快推进《珠中江区域紧密合作规划》的编制工作。加强与广佛珠中“十二五”规划期间的经济社会发展战略、重大产业布局、城市总体规划、土地利用总体规划及有关重大专项规划的衔接。积极推进以交通一体化为重点的基础设施一体化。以落实年票互认、饮用水同网、通信一体化为重点，加快推进“珠中江”经济圈基本公共服务一体化。积极推进珠中江及澳门旅游合作，打造珠三角西部统一旅游市场。积极推进珠三角产业布局一体化，力争启动3-5个实质性产业协作项目。

　　加快推进滨江新区和市区一体化建设。积极拓展城市空间，优化市区战略布局。投资23亿元，加快滨江新区启动区13项重点基础设施工程建设，年内建成雁山互通立交。建设新市民广场、天沙河绿化景观带、丰乐山顶公园和市区旗杆石垃圾处理场等。推进实施全市综合交通一体化规划。加大公共交通设施改造力度，完成里村大道建设，重点抓好大洞大桥、东华大桥、胜利大桥、礼东大桥、胜利南路、白沙大道东和市区公共停车场的建设，以及金瓯路、江睦路、北环路和跃进路的扩宽改造，提高生态宜居水平。落实《推进市区一体化建设实施意见》，理顺三区在自来水、清洁卫生、生活垃圾、有线电视、公交等方面的价格差，实现同城一体化。

　　扎实推进节能减排和节约集约用地。抓住先行先试机遇，努力在节约集约发展上实现新突破。编制发展低碳城市战略规划，探索建设低碳城市。严格执行节能问责制和“一票否决”制。严格实行主要污染物排放总量控制制度，强力推进淘汰落后产能工作。推广建筑节能，抓好半导体照明产品示范工程。扎实推进循环经济、清洁生产和资源综合利用工作。加快推进城镇污水处理设施建设，确保文昌沙水质净化厂二期、江海污水处理厂首期建成并投入运行，全面完成中心镇污水处理设施建设任务，新增污水日处理能力18万吨以上。完善土地储备机制，开发利用园地山坡地补充耕地1.5万亩，有计划推进围填海，加快实施新会银湖湾区域和台山广海湾建设用海规划，保障经济社会发展和重点项目用地需求。坚决处置闲置地，整合零散地、低效地，提高土地执法监管水平。加强矿政管理，做好地质灾害防治。

　　四、着力促进农民稳定增收，扎实推进新农村建设

　　大力发展农业农村经济。今年全市各级财政预算用于“三农”支出35.3亿元，比上年增长21.03%。制定农业产业发展规划（2010-2020年），优化农业产业布局。办好第二届农业博览会。大力发展海洋经济和现代渔业。培育新型农业经营主体，提高产业化水平。加快现代农业综合示范基地和人才创业农业示范基地建设，大力发展品牌农业和农业标准化示范区，高标准整治农田2.3万亩。加快农产品质量安全监测体系建设，推行食用农产品标识管理。抓好农村土地承包经营权流转试点建设。稳步推进集体林权制度改革。加快华侨农场发展。

　　大力发展农业现代服务体系。加快供销合作社改革发展和新农村现代流通服务网络建设，构建现代农业生产资料经营服务、农村日用消费品经营、农副产品购销、再生资源回收利用等网络体系。实现“农村信息直通车”覆盖所有行政村和农村经济合作组织，提高“三农”信息服务水平。探索建立农业政策性保险制度和农业再保险机制、巨灾风险分散机制，进一步拓宽农业政策性保险种类和范围，扩大政策性水稻种植保险覆盖面。引进多种形式的新型农村金融机构，将农信社发展为农村商业银行。扩大农村有效担保物范围，开展宅基地、土地承包权、农村住房抵押借款试点。扶持有条件的农民专业合作经济组织开展信用合作。加强动植物疫病防控。

　　大力开展宜居村庄建设。年内村庄规划编制完成率达到50%、村庄整治任务完成率达到40%。加快推进农村“五改”和“脏乱差”治理，完善“户集、村收、镇运、县（市）处理”的农村生活垃圾收集处理和镇村生活污水处理机制。加快农村饮水安全工程建设。大力发展农村沼气，推广应用“养殖—沼气—种植”生态农业模式。

　　大力推进扶贫工程。深入推进“千企扶千村”、“万人扶千户”工程，完善企业、善心人士与贫困村、贫困户对接服务平台。在所有行政村全面开通远程可视招聘系统，年内培训农村青年和农村富余劳动力不少于4万人，实现转移就业不少于3万人。采取“规划到户、责任到人”、“一村一策、一户一法”等综合扶贫措施，确保到2011年底完成省下达我市的扶贫任务。

　　五、着力改善社会民生，深入推进创建全国文明城市工作

　　各级财政投入49亿元改善民生，比上年增长19.5%。大力营造全民参与“创文”的氛围，广泛开展“办实事解民忧”活动，发动广大市民群众积极参与“创文”并享受“创文”成果。

　　加快建立覆盖城乡的社会保障体系。全市社会保障体系建设财政投入6.44亿元，比上年增长37.35%。扩大新型农村基本养老保险试点，将全市15%以上的农民纳入基本养老保险范围。开展居民基本医疗保险制度城乡一体化和基本医疗保险门诊统筹试点。落实城镇职工失业、工伤、生育保险市级统筹，推进医疗保险市级统筹，探索珠中江医疗保险一体化机制。推进工资集体协商制度，健全欠薪预警保障制度，建立劳动保障与银行发放工资联网机制。加强企业退休人员社区管理，提高社区管理服务水平。

　　积极稳定和促进就业。新增就业4.5万人。实施创业带动就业计划，建立市级创业带动就业孵化基地，举办青年创业项目设计大赛。提高职业培训能力，实施技能就业、素质就业计划，培养适应我市产业升级所需的技能人才。落实高校毕业生就业创业扶持政策，新建高校毕业生见习基地60家。深入开展城乡就业援助行动，解决好困难群体的就业难问题。加快珠中江人力资源服务体系一体化建设。

　　巩固教育强市创建成果。各级财政预算教育投入23.38亿元，比上年增长14.77%。大力推进义务教育资源均衡布局、合理配置，建立和完善义务教育均衡发展的监督制度。切实提高义务教育和普通高中教育质量，创新发展职业教育，鼓励发展民办教育，重视学前教育和特殊教育。新增4个教育强镇(街)。提升五邑大学和江门职院办学水平。实施中小学校舍安全工程。加强未成年人思想道德教育。加强教师队伍建设，进一步落实中小学绩效工资和教师工资福利待遇“两相当”工作。

　　强化公共卫生服务。落实新医药卫生体制改革方案，执行国家基本药物制度，在条件成熟的公办社区卫生服务机构及基层医疗卫生机构，探索开展基本药物零差率销售。推进农村三级医疗卫生服务网络和城市社区卫生服务中心规范化建设，鼓励大医院与社区医疗机构挂钩。完善医疗应急体系，做好甲型流感等重大传染疾病防控。加快中医药名市建设和民营医疗机构发展。

　　加强城市综合管理。完善城市综合管理重心下移工作机制。建立健全对扰民问题的及时处理机制。每季度开展一次以城市综合管理为重点的满意度问卷社会调查，并在媒体上公布，不满意度高的相关部门要作公开回应。推进平安和谐社区建设，全市“六好”平安和谐社区达80%以上。完善社会治安防控体系，以加强治安巡逻、整治网吧、打击车辆盗窃和公交车盗窃为重点，依法打击各类违法犯罪活动。建立社会治安满意度与公安局及基层派出所班子成员考核挂钩机制和“大走访”长效工作机制，开展“民警进千家走万户”活动。加大卫生死角、机动车尾气、油烟废气的治理力度。启动“健康城市”创建工作。发挥镇（街）综治信访维稳中心作用，加强基层信访工作，完善社会矛盾纠纷调处机制，实现社会管理信息共建共享。健全公共突发事件应急机制。严格落实安全生产监管责任和企业安全生产主体责任，抓好重点行业和领域的治理，推动企业安全生产标准化建设，持续遏制较大以上事故发生。

　　做好文化、体育、人口计生等工作。整合资源加大侨乡品牌宣传力度。组织策划“今日江门”宣传推介活动，打响珠三角一流投资热土、广东一流宜居城市、国内一流旅游胜地、世界一流摩托名城品牌。加快文化产业发展，推进动漫影视产业园建设；办好2010中国（江门）侨乡华人嘉年华暨旅游节和圭峰庙会；发挥五邑籍港台明星的效应。开展“双百”优秀务工人员评选活动。推进城乡青年中心和“义工”服务站建设，创建全国公共文化服务示范区。改造提升五邑华侨华人博物馆布展水平。加强文化遗产保护，建立保护和传承非物质文化遗产长效机制。积极开展文化下乡、进社区活动，实施农村电影放映工程、农家书屋和文化信息资源共享工程。加强新闻出版、广播电视和文化市场管理。发展慈善公益事业。普及全民健身设施建设，广泛开展群众性体育活动。提高竞技体育水平，积极迎战亚运会和省运会。做好人口和计划生育工作，稳定低生育水平，提高出生人口质量。落实妇女儿童发展规划，维护妇女儿童权益。发展残疾人事业。加强国防教育，完善国防动员体系，落实“双拥”和优抚安置工作。继续做好民族、宗教、侨务、外事、港澳、对台、老龄、人防、统计、审计、档案、方志、气象、红十字会等工作和对汶川灾区援建后续工作。

　　六、着力提高服务满意度，进一步加强服务型政府建设

　　做好今年各项工作，是对政府施政能力的检验。我们要主动适应形势发展的新要求，迎合人民群众的新期盼，切实加强政府自身建设，更好地为发展服务，为广大人民群众服务。

　　大力提高行政效能。深入开展机关作风建设年活动，重点解决“首办效率低”和“执罚随意性大”这两个突出问题。建立首办（首问）有效统计、跟踪改进机制和不满意事项有效投诉领导问责制，对涉民涉企部门每半年开展一次以首办（首问）为重点的满意度调查，调查结果纳入作风建设考评内容。进一步规范执法自由裁量权，对一般性问题以“从宽、从轻、就低”为原则，实行主要领导把关，制订具体的执罚操作办法，解决执罚随意性大的问题。扎实推进ISO质量管理工作。大力精简会议，压减文件数量和篇幅，大兴调查研究之风。强化绩效考评，提高工作效能。

　　进一步提高依法行政、科学决策水平。认真落实人大及其常委会各项决议决定，坚持向人大及其常委会报告工作，自觉接受监督。配合人大开展好“人大代表约见市长”、“人大代表约见局长”活动。加强与人民政协的联系及工作通报，支持政协委员发挥参政议政作用。提高议案、建议和提案办理质量。健全政府信息公开、规范性文件管理、行政执法责任制、行政复议、投诉申诉依法办理等制度。抓好“十二五”规划的研究和编制。坚持经济形势分析联席会议制度、经济社会发展顾问咨询制度、重大事项公开征集意见和合法性审查制度，完善行政决策纠错机制。广泛听取各民主党派、工商联、无党派人士意见，支持工会、共青团和妇联等人民团体更好地发挥作用。

　　全面提高廉政建设水平。扎实推进惩治和预防腐败体系建设，进一步从源头上防治腐败。严格执行公务购车和用车经费、会议经费、公务接待费用、机关出国(境)费用、办公经费预算零增长和一般性支出预算压缩5%的要求。开展公务卡结算方式试点工作。建设农村党风廉政信息平台。在市直机关试行廉政公积金制度。探索建立对贪污腐败“零容忍”的检举监督机制。继续加大案件查处力度，严惩腐败分子，以反腐倡廉的实际成效取信于民。

　　各位代表，新的一年，困难和挑战考验着我们，责任和使命激励着我们。让我们在省委、省政府和江门市委的正确领导下，高举中国特色社会主义伟大旗帜，深入贯彻落实科学发展观，紧紧依靠全市人民，解放思想，同心同德，开拓进取，扎实工作，努力实现江门侨乡新的更大的发展，向全市人民交出一份更加满意的答卷！
